# A patient with typical clinical features of mitochondrial encephalopathy, lactic acidosis and stroke-like episodes (MELAS) but without an obvious genetic cause: a case report

**DOI:** 10.1186/1752-1947-3-77

**Published:** 2009-10-15

**Authors:** Khaled K Abu-Amero, Hesham Al-Dhalaan, Saeed Bohlega, Ali Hellani, Robert W Taylor

**Affiliations:** 1 Molecular Genetics Laboratory, College of Medicine, King Saud University, Riyadh, Saudi Arabia; 2 Neuroscience Department, King Faisal Specialist Hospital and Research Centre, Riyadh, Saudi Arabia; 3 PGD Laboratory, Saad Specialist Hospital, Al-Khobar, Saudi Arabia; 4 Mitochondrial Research Group, The Medical School, Newcastle University, Newcastle upon Tyne, UK

## Abstract

**Introduction:**

There are currently 23 missense point mutations and one 4 basepair deletion spanning different mitochondrial genes associated with mitochondrial encephalopathy, lactic acidosis and stroke-like episodes (MELAS). The spectrum of mitochondrial DNA mutations in Arab patients with MELAS is largely unknown.

**Case presentation:**

A standard clinical examination was carried out on a 34-year-old Saudi woman showing clinical features of MELAS. Fresh frozen muscle tissue was subjected to enzyme histochemical analysis. DNA was extracted from her leukocytes and muscle tissue, and the full mitochondrial genome was screened for base substitution mutations and deletions. Additionally, we screened the polymerase gamma-1 nuclear gene for mutations. The patient was negative for the most common m.3243 A>G MELAS mutation. Sequencing the full mitochondrial genome did not reveal any known or potentially pathogenic sequence changes. The polymerase gamma-1 gene was also free from mutations.

**Conclusion:**

The clinical picture described here typically fits that observed in patients with MELAS or mitochondrial stroke-like events, but mutations in recognized genes (mitochondrial DNA and polymerase gamma-1 gene) were absent. We report the case of a patient with typical clinical features of MELAS, but without an obvious genetic cause.

## Introduction

Mitochondrial encephalopathy, lactic acidosis and stroke-like episodes (MELAS) is a mitochondrial disorder typically associated with seizures and/or dementia with elevated levels of cerebrospinal fluid (CSF) lactate. It is the most common maternally inherited mitochondrial disease, with an A>G mutation at position 3243 in the *MTTL1 *tRNA^Leu(UUR) ^of the mitochondrial DNA (mtDNA) gene responsible for more than 80% of cases [1]. Currently, there are 23 missense point mutations and one 4 base pair (bp) deletion spanning different mitochondrial genes that have been previously reported in association with MELAS (MITOMAP: http://www.mitomap.org). The spectrum of mtDNA mutation in Arab patients with MELAS is largely unknown, although one study has reported a novel *MTTL2 *gene mutation in a Saudi boy with typical clinical features of MELAS [2], highlighting the fact that some MELAS patients who fulfill the traditional clinical criteria for MELAS could be negative for the most common m.3243A>G mutation in this population. In such cases, the patient should be screened for secondary mtDNA changes such as multiple mtDNA deletions and for mutations in the mtDNA polymerase gamma (*POLG1*) nuclear gene. Mutations in the *POLG1 *gene have been implicated in a patient with MELAS [3]. In this study, we describe a female patient with: i) typical clinical features of MELAS; ii) absence of the MELAS m.3243A>G common mutation or any other obvious mtDNA point mutation; and iii) absence of any *POLG1 *mutation(s).

## Case presentation

We have investigated the case of a 34-year-old Saudi woman who presented with clinical symptoms suggestive of MELAS. She was otherwise normal, able to finish college, had started work as a teacher and had married at the age of 22. She was well until age 24 when she started to have generalized tonic clonic seizures, which were successfully controlled with anti-epileptic drugs. She lost her vision at the age of 27 and then became deaf, and at the age of 32, she developed right-sided weakness. At the age of 33, she was admitted to hospital with abdominal pain and tenderness, hypoactive bowel sound, vomiting, dehydration and hypokalemia. The symptoms were highly suggestive of pseudo-intestinal obstruction, which improved with conservative measures. She reported no family history of a similar condition and her parents confirmed this. She was underweight with a weight ranging from 33 to 34.5 kg and a height of 152 cm. Her serum lactate was elevated at 3.8 mM (normal < 2 mM) as was her CSF lactate at 4.5 mM.

Work-up initially showed a left occipital lobe infarction followed by progressive decline in visual acuity and hearing. Her seizures were controlled for 18 months with lamotragine, but subsequent medication was discontinued gradually. She then developed an episode of acute visual loss, generalized tonic clonic seizures and status epilepticus, which was complicated by an aspiration pneumonia for which she required admission to the intensive care unit (ICU). Her general condition deteriorated rapidly, requiring ventilation support and a gastrostomy tube for feeding. She stayed in the ICU for approximately 2 months during which she became very wasted, spastic, mute, deaf, and blind. Cornea reflexes were present and gag reflex absent.

**Figure 1:**
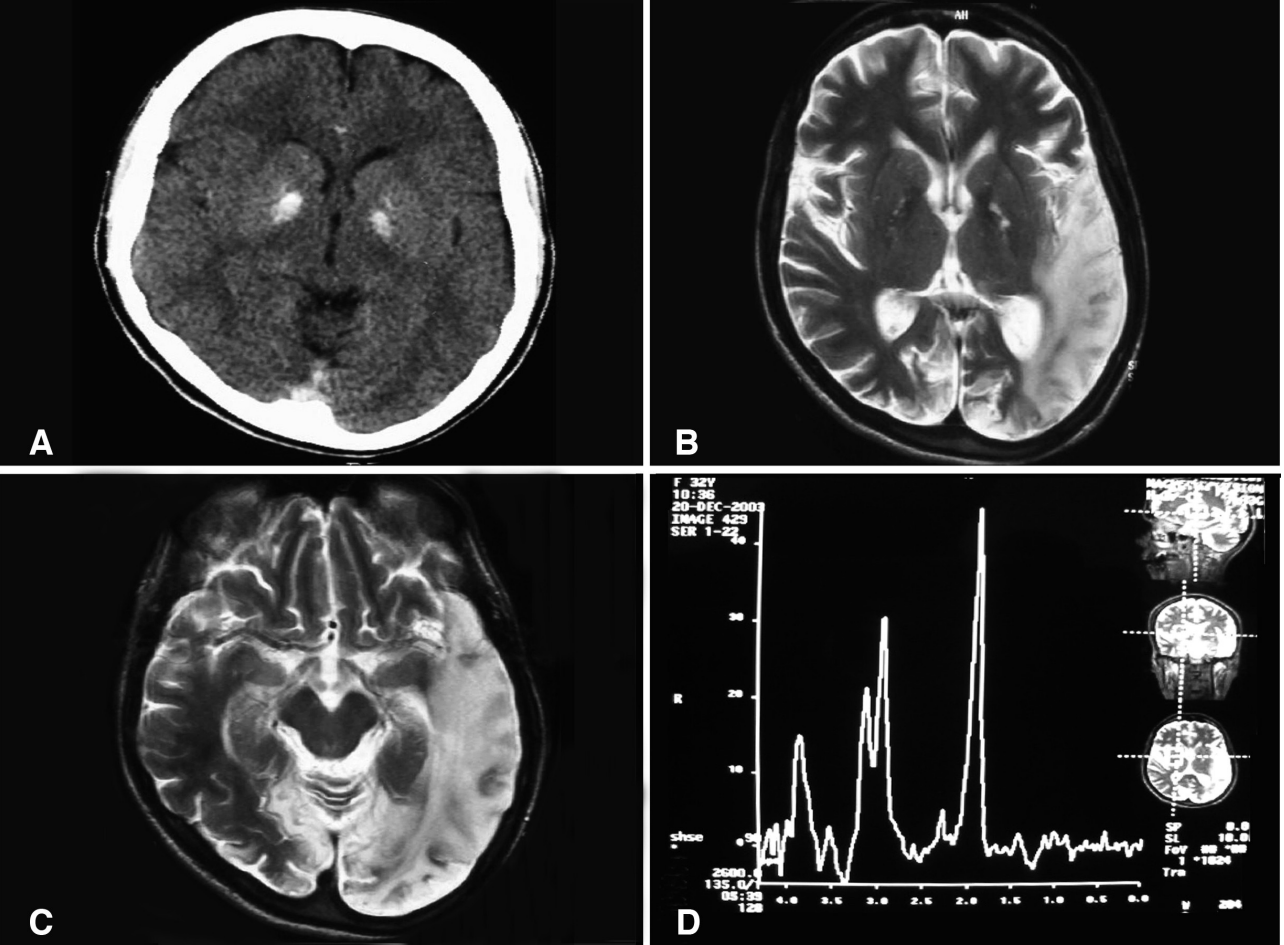
**(a) A computed tomography brain scan showing bilateral basal ganglia calcification; the cerebellum shows prominent folia indicating mild cerebellar atrophy**. (b) Axial T2 brain magnetic resonance image scan showing left temporo-parieto occipital ischemic lesion. (c) Axial T2 brain magnetic resonance image scan showing the extension of the parietal temporal region to the occipital lobe, and also showing a right occipital lesion. (d) Magnetic resonance spectroscopy showing inversion of J-coupling phenomenon at 1.3 ppm, indicating lactate peak.

A computed tomography (CT) brain scan showed bilateral basal ganglia calcification and mild cerebellar atrophy (Figure 1a). Her axial T2 brain magnetic resonance imaging (MRI) scan showed a left temporo-parieto occipital ischemic lesion (Figure 1b), which extended beyond the typical vascular territory of the left middle cerebral artery (Figure 1c). Magnetic resonance spectroscopy (MRS) showing inversion of the J-coupling phenomenon at 1.3 ppm indicating a lactate peak (Figure 1d).

**Figure 2:**
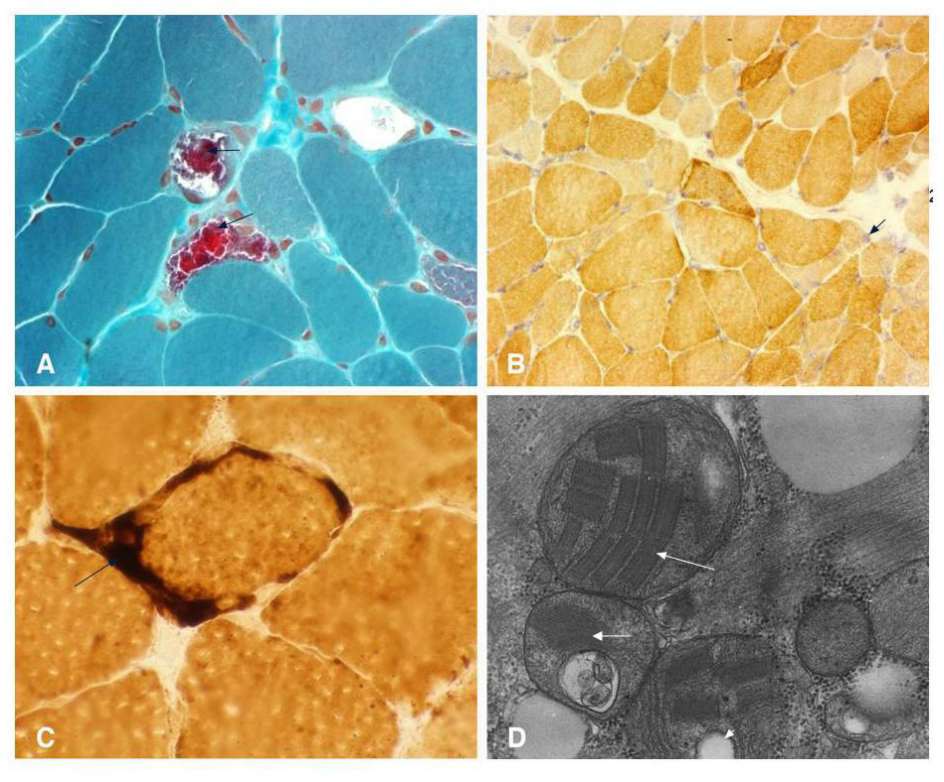
**(a) Modified Gomori trichrome stain showing several ragged red fibers (arrowhead)**. (b) Cytochrome *c *oxidase stain showing Type-1 lightly stained and Type II fibers, darker fibers, and a few fibers with abnormal collections of mitochondria (arrowhead). Note cytochrome *c *oxidase negative fibers as usually seen in mitochondrial encephalopathy, lactic acidosis and stroke-like episodes (MELAS). (c) Succinate dehydrogenase staining showing a few ragged blue fibers and intense staining in the mitochondria of the blood vessels (arrow). (d) Electron microscopy showing abnormal collection of mitochondria with paracrystalline inclusions (arrowhead), osmiophilic inclusions (large arrowhead) and mitochondrial vacuoles (small arrowhead).

The patient consented to a muscle biopsy, which was subjected to enzyme histochemical analysis as previously described [4]. Modified Gomori trichrome stain revealed ragged red fibers (Figure 2a) and cytochrome *c *oxidase (COX) staining showed abnormal accumulation of brownish staining indicating accumulation of mitochondria (Figure 2b). Succinate dehydrogenase (SDH) staining showed a few ragged blue fibers and intense staining in the mitochondria of the blood vessels, a feature commonly seen in MELAS cases (Figure 2c).

Electron microscopy showed an abnormal collection of mitochondria with paracrystalline inclusions, osmiophilic inclusions and mitochondrial vacuoles (Figure 2d). While analysis of respiratory chain enzymes in the muscle was not possible, we decided to pursue the underlying genetic defect based on the mitochondrial histochemical changes we observed, which were diagnostic of an underlying mtDNA genetic abnormality.

Skeletal muscle DNA was used to determine the sequence of the entire mtDNA coding region, but we did not detect any reported pathological or potentially pathological sequence changes.

Recent findings of mutations in the nuclear-encoded gene encoding the catalytic subunit of the mtDNA polymerase gamma-1 (*POLG1*) gene [3] promoted us to screen this gene for mutations but no pathogenic mutation was identified. The absence of mutations within the mtDNA and the *POLG1 *gene prompted further investigation into secondary mtDNA changes.

## Discussion

The m.3243A>G common MELAS mutation is responsible for more than 80% of reported MELAS cases [1]. Most of these reports came from western countries. However, more than 85% of the DNA samples sent to the Molecular Diagnostic Laboratory at King Faisal Specialist Hospital from Arab patients with typical clinical features of MELAS were negative for the m.3243A>G common MELAS mutation (personal observation). In support of this, we previously reported a novel *MTTL2 *gene mutation in a Saudi boy with typical clinical features of MELAS [2]. Additionally, we have reported a different pattern of mutations in a group of Arab patients with typical clinical features of Leber's Hereditary Optic Neuropathy (LHON). In western countries, the three primary LHON mutations were detected in > 90% of LHON cases, while only 17% of Arab LHON patients had one of the three LHON mutations. The remaining 83% had different mtDNA mutations or other types of mitochondrial abnormalities [5]. These findings highlight a possible variability in the mtDNA mutation pattern among patients from different ethnicities with the same disease. This observation, and the fact that mitochondrial disorders are clinically variable disorders, make it a challenge to draw reasonable phenotype-genotype correlations. Having said that, the typical clinical features observed among Arab patients with MELAS are not different from those observed in western countries [6] and the variability is only limited to the spectrum of mutations detected. Our previous findings in the LHON study [5] support this hypothesis.

The patient described here had clinical symptoms suggestive of MELAS. This was supported by her neuroimaging presentation, histochemical analysis, high serum and CSF lactate levels and electron microscopic results from her muscle tissues. Genetics analysis of her DNA indicated that she was negative for the m.3243A>G common MELAS mutation and screening of her entire mtDNA genome did not reveal any reported pathogenic or potentially pathogenic mtDNA mutation. When we screened the entire mitochondrial genome of this patient, we ignored any DNA variant not resulting in an amino acid change (synonymous nucleotide changes) and this is the current practice in medical genetics. Nowadays, however, numerous DNA variants that are not associated with an amino acid change have been shown to have deleterious effects [7]. Because of this, we could possibly have missed the causal mtDNA base substitution. Recent findings of mutations in the nuclear-encoded gene encoding the catalytic subunit of the mtDNA polymerase gamma (*POLG1*) [3], prompted us to screen this gene for mutations but no pathogenic mutations were identified after sequencing the entire gene. We cannot of course ignore the contribution of other nuclear genes encoding proteins responsible for stability and structural components of the mtDNA or to the oxidative phosphorylation (OXPHOS) machinery being involved in the pathogenesis of MELAS-like disease.

## Conclusion

We report a patient with typical clinical features of MELAS but without an obvious genetic cause.

## Abbreviations

COX: cytochrome *c *oxidase; CSF: cerebrospinal fluid; CT: computed tomography; ICU: intensive care unit; MELAS: mitochondrial encephalopathy, lactic acidosis and stroke-like episodes; MRI: magnetic resonance imaging; MRS: magnetic resonance spectroscopy; mtDNA: mitochondrial DNA; OXPHOS: oxidative phosphorylation; PCR: polymerase chain reaction; *POLG1*: polymerase gamma-1; SDH: succinate dehydrogenase.

## Competing interests

The authors declare that they have no competing interests.

## Authors' contributions

KKA was in charge of design, analysis of data and overall supervision of the study. AH performed the technical aspects of the study, PCR and sequencing. RWT performed the sequencing of the *POLG1 *and wrote part of the manuscript. SB and HD were responsible for recruiting the patient and the detailed clinical evaluation.

## Consent

Written informed consent was obtained from the parents of the patient for publication of this case report and any accompanying images. A copy of the written consent is available for review by the Editor-in-Chief of this journal.
